# Supplementary material for: Impact of coronavirus disease 2019 on cancer care: How the pandemic has changed cancer utilization and expenditures
Source: PLoS One. 2024 Feb 8;19(2):e0296808. doi: 10.1371/journal.pone.0296808 (PMC10852310; doi:10.1371/journal.pone.0296808)
Supplement: S1 Table — (DOCX) [file pone.0296808.s001.docx]

**S1 Table. Comparisons of average of admission, average of admission days per person**

| Year | Average of admission per person | Average of admission days per person |
| --- | --- | --- |
| 2014 | 1.34 | 13.61 |
| 2015 | 1.35 | 13.73 |
| 2016 | 1.37 | 13.52 |
| 2017 | 1.40 | 13.37 |
| 2018 | 1.43 | 13.35 |
| 2019 | 1.45 | 13.46 |
| 2020 | 1.42 | 13.39 |
