# Supplementary figures and images for: Impact of coronavirus disease 2019 on cancer care: How the pandemic has changed cancer utilization and expenditures
Source: PLoS One. 2024 Feb 8;19(2):e0296808. doi: 10.1371/journal.pone.0296808 (PMC10852310; doi:10.1371/journal.pone.0296808)

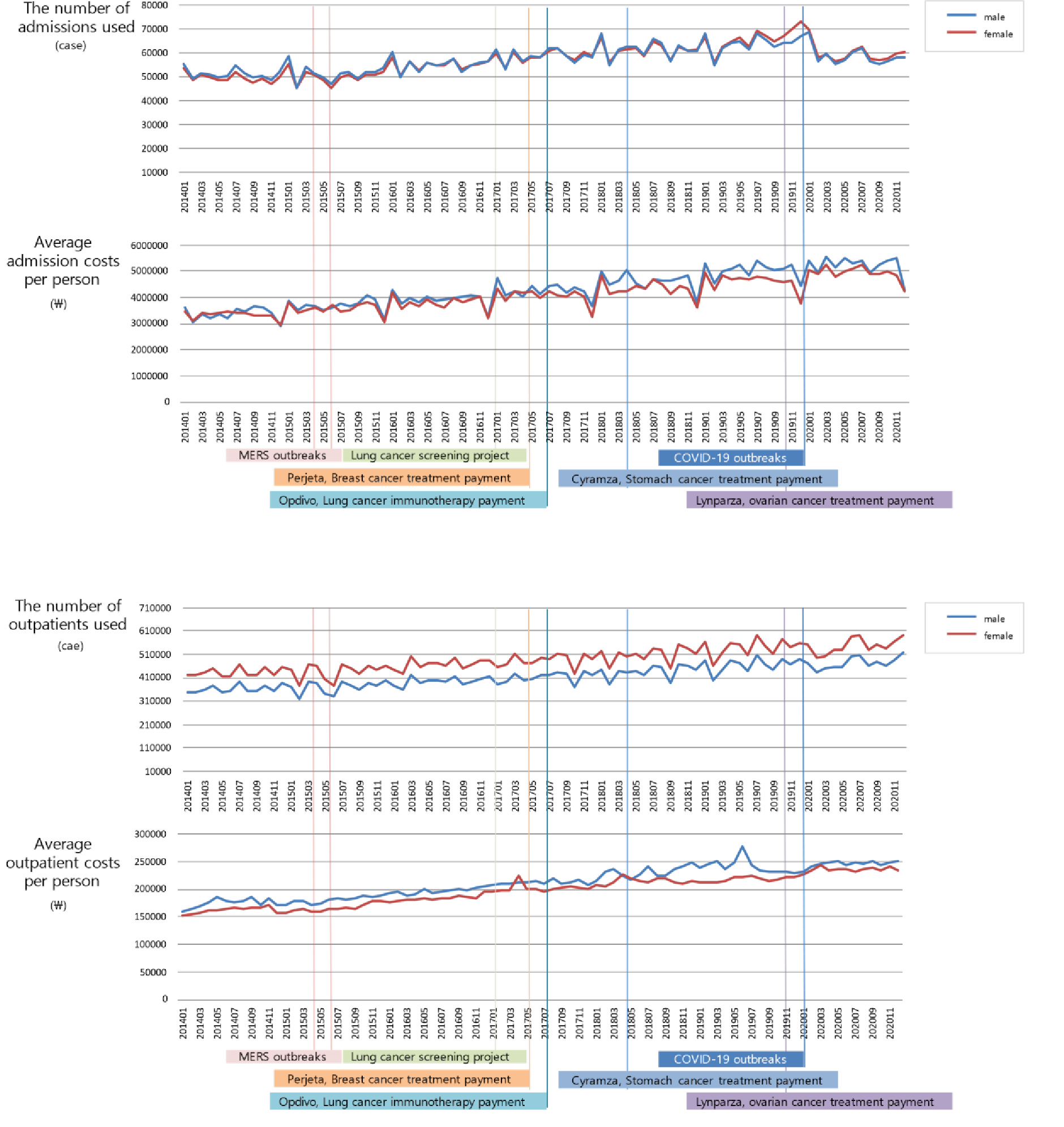

Supplement: S1 Fig — (TIF) [file pone.0296808.s002.tif]

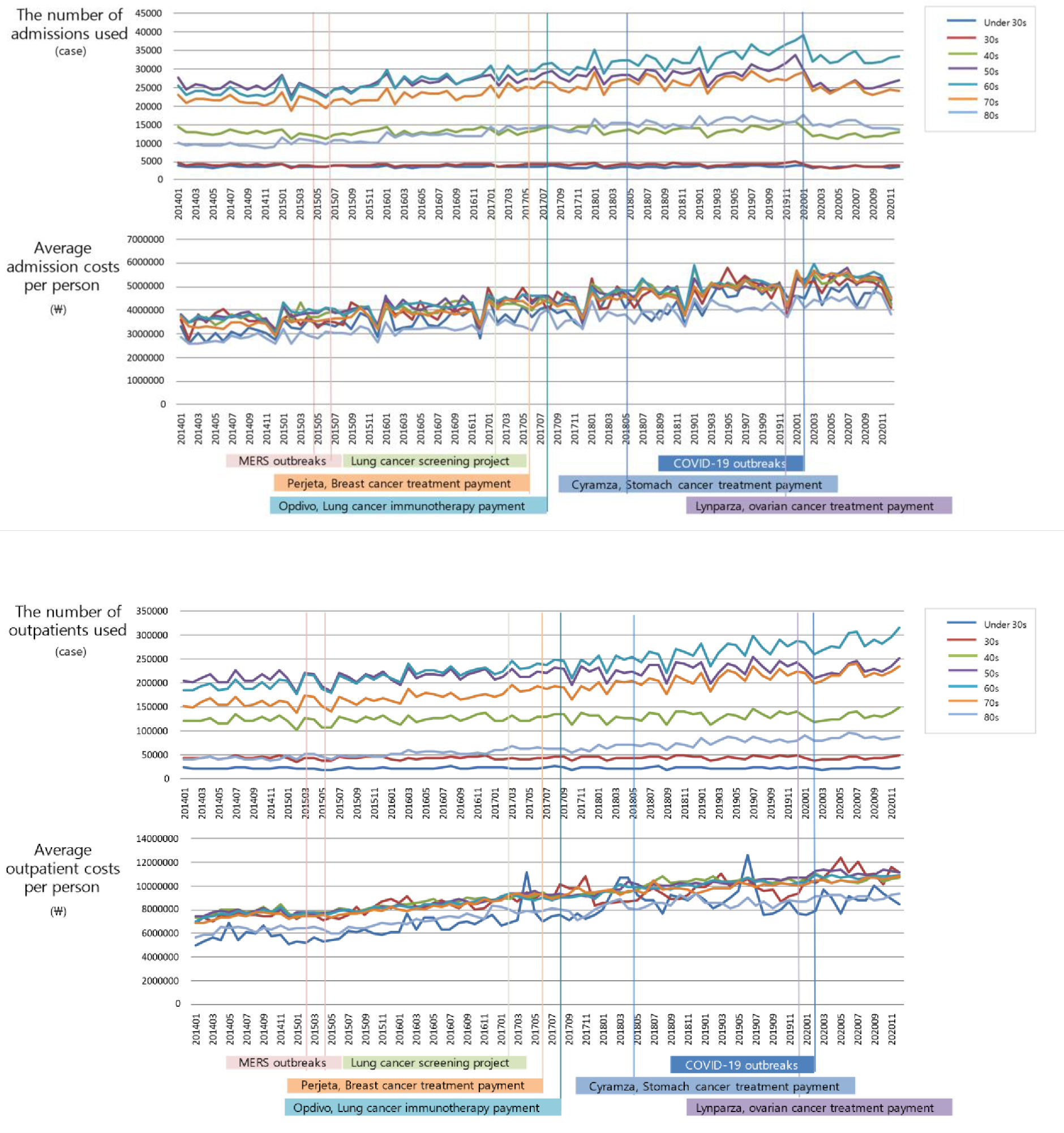

Supplement: S2 Fig — (TIF) [file pone.0296808.s003.tif]

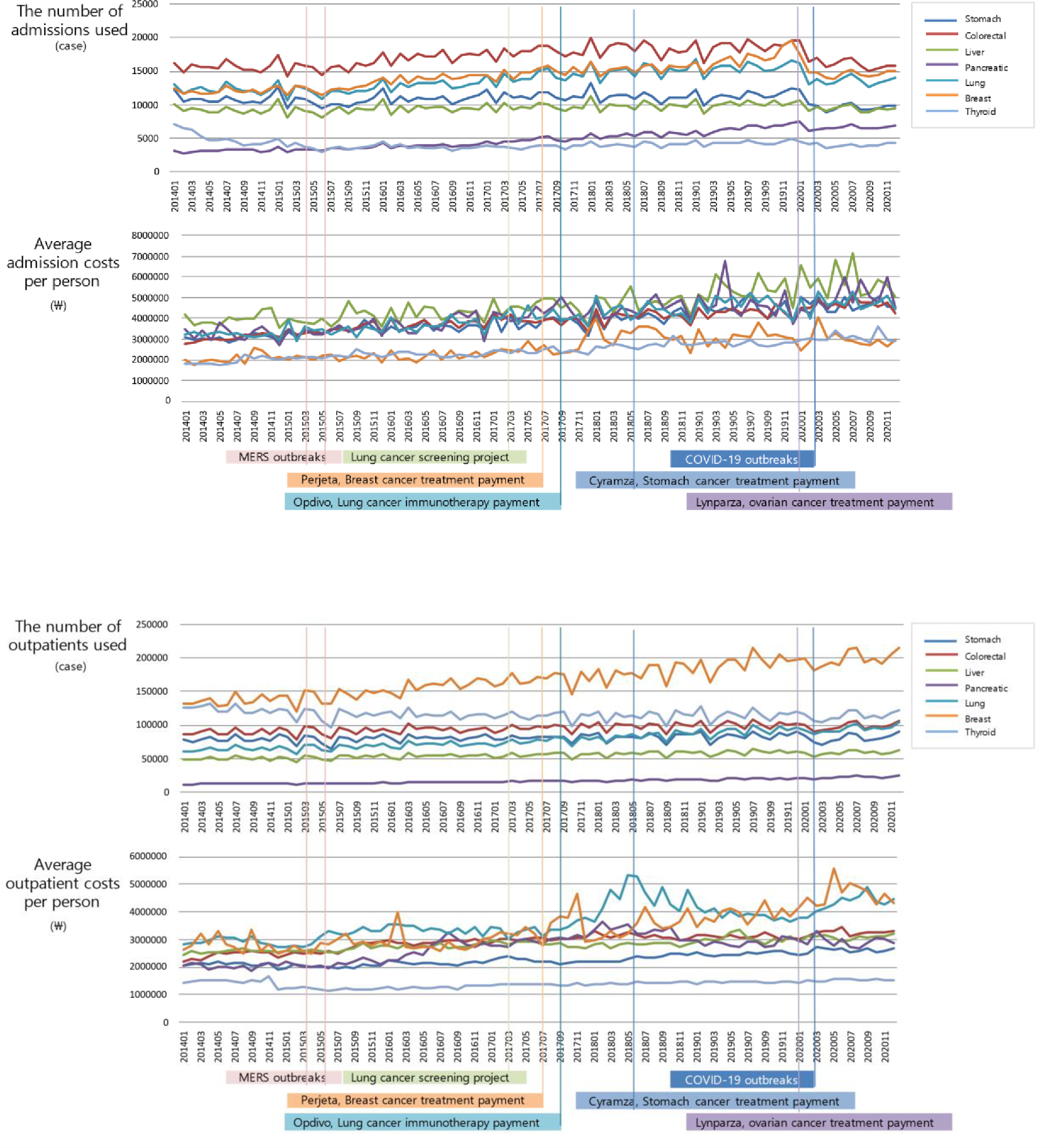

Supplement: S3 Fig — (TIF) [file pone.0296808.s004.tif]

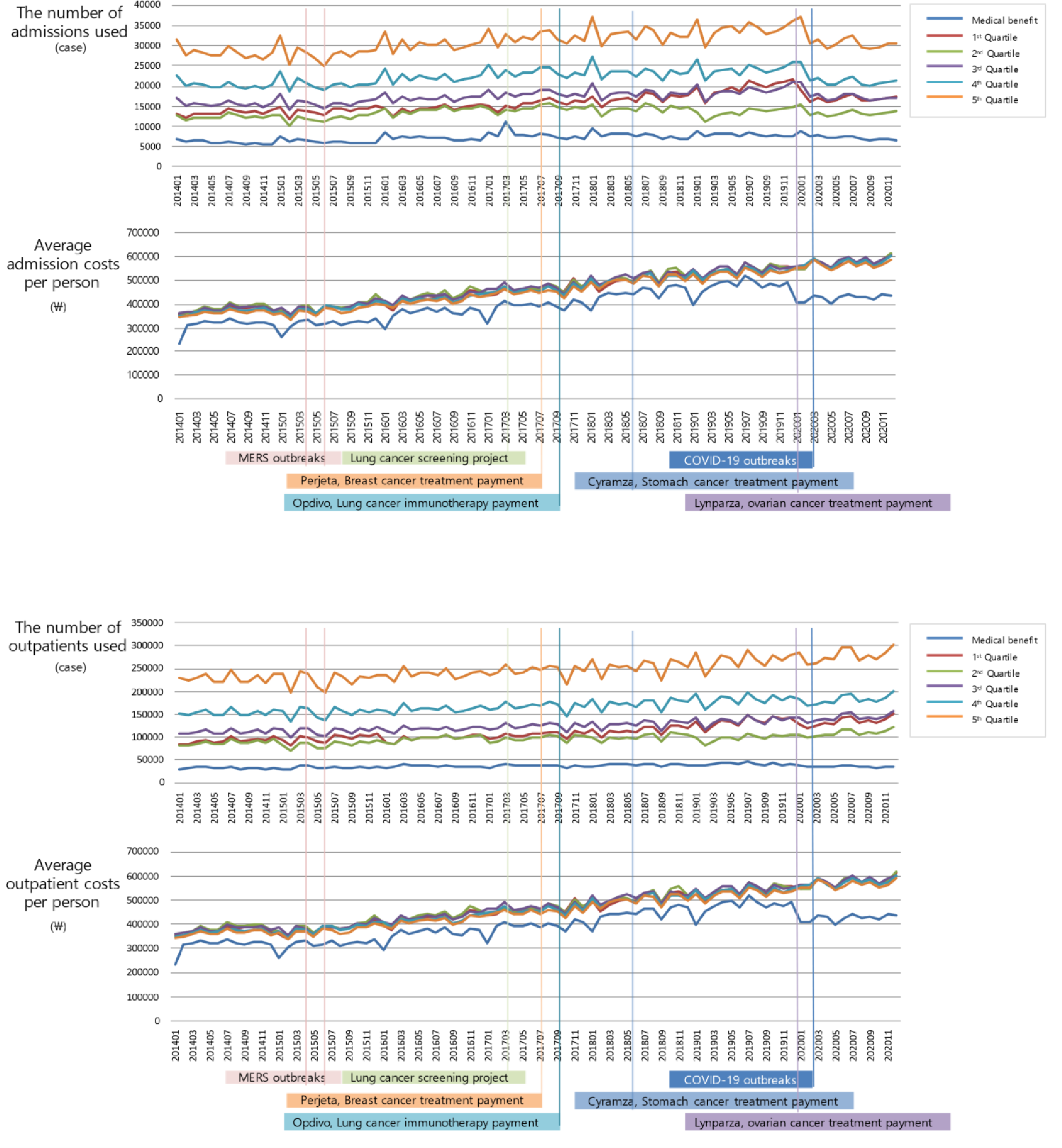

Supplement: S4 Fig — (TIF) [file pone.0296808.s005.tif]

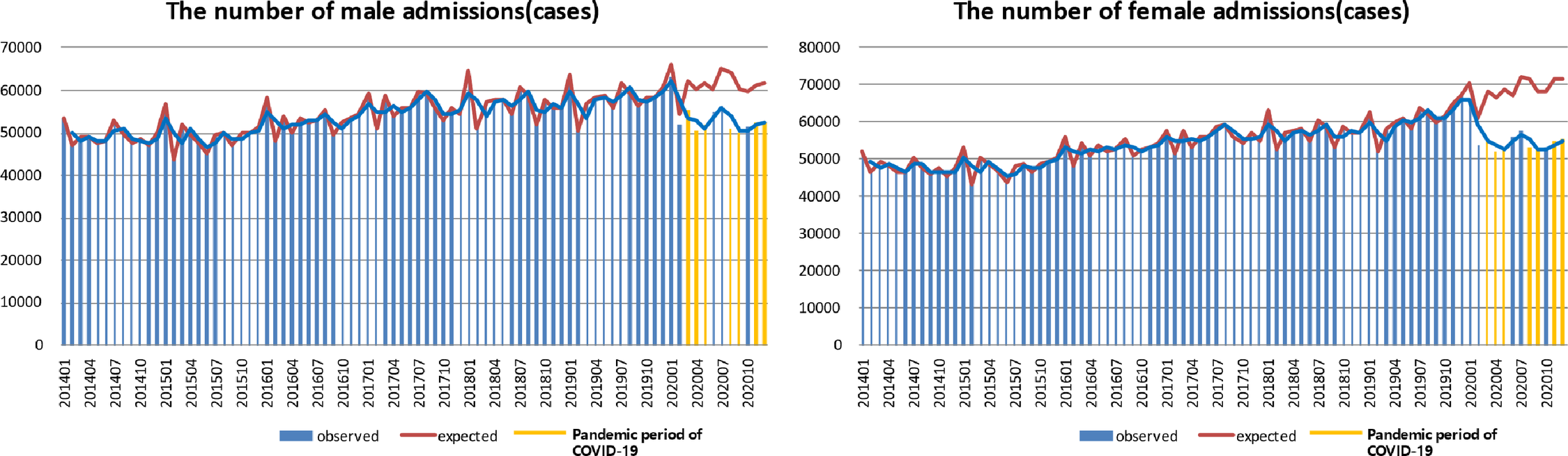

Supplement: S5 Fig — (TIF) [file pone.0296808.s006.tif]

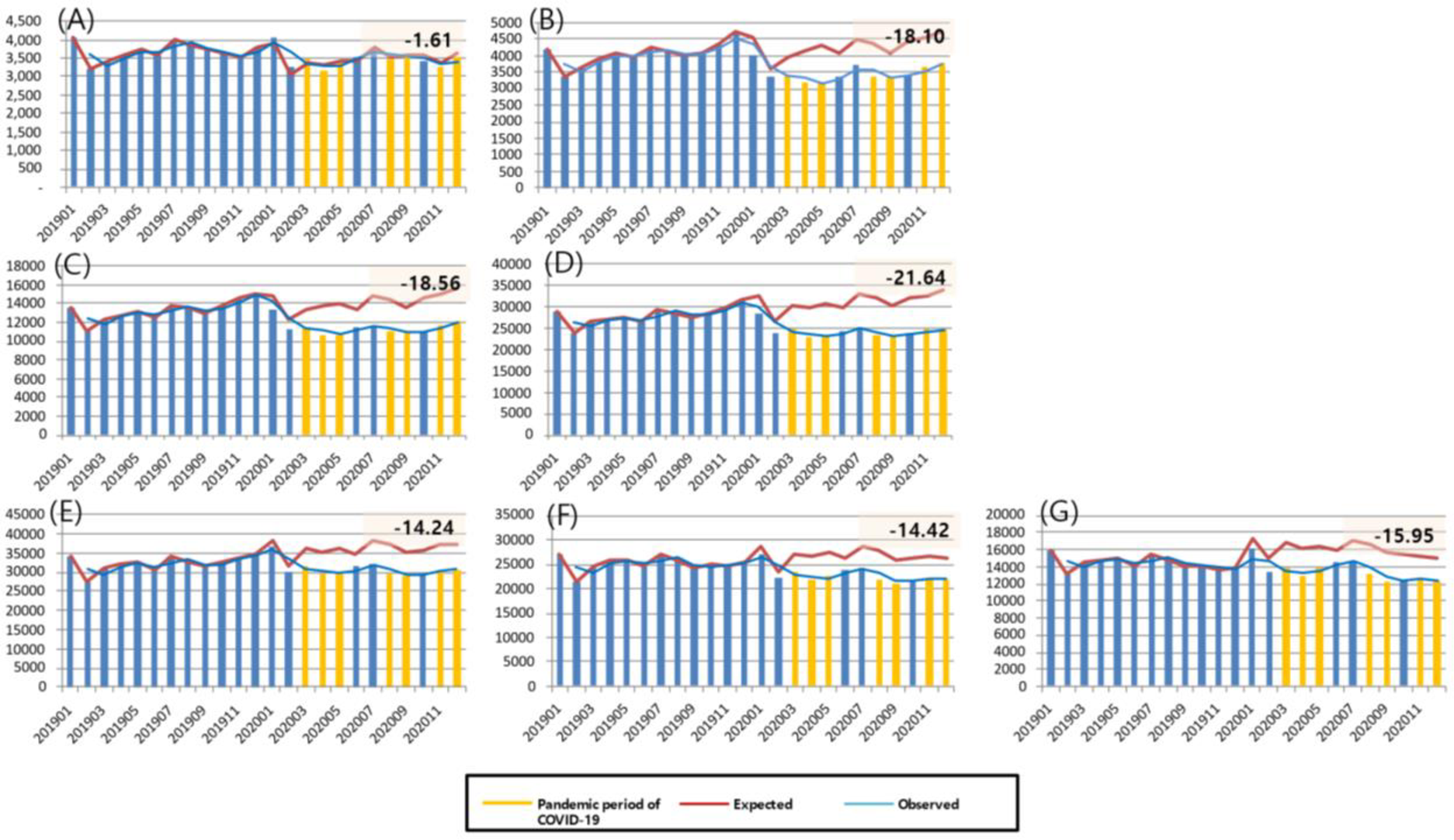

Supplement: S6 Fig — (A) Under 30s, (B) 30s, (C) 40s, (D) 50s, (E) 60s, (F) 70s, (G) 80s. (TIF) [file pone.0296808.s007.tif]

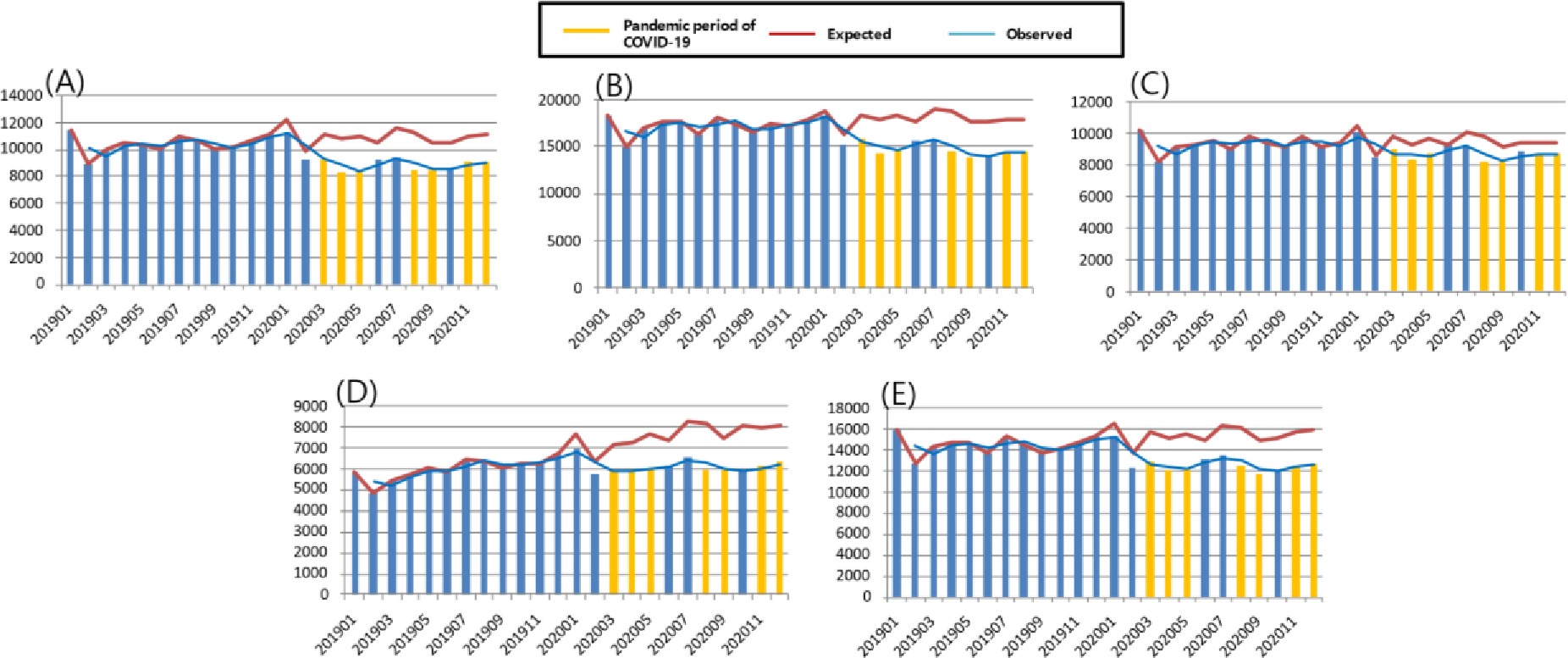

Supplement: S7 Fig — (A) Stomach admission, (B) Colon admission, (C) Liver admission, (D) Pancreatic admission, (E) Lung admission. (TIF) [file pone.0296808.s008.tif]

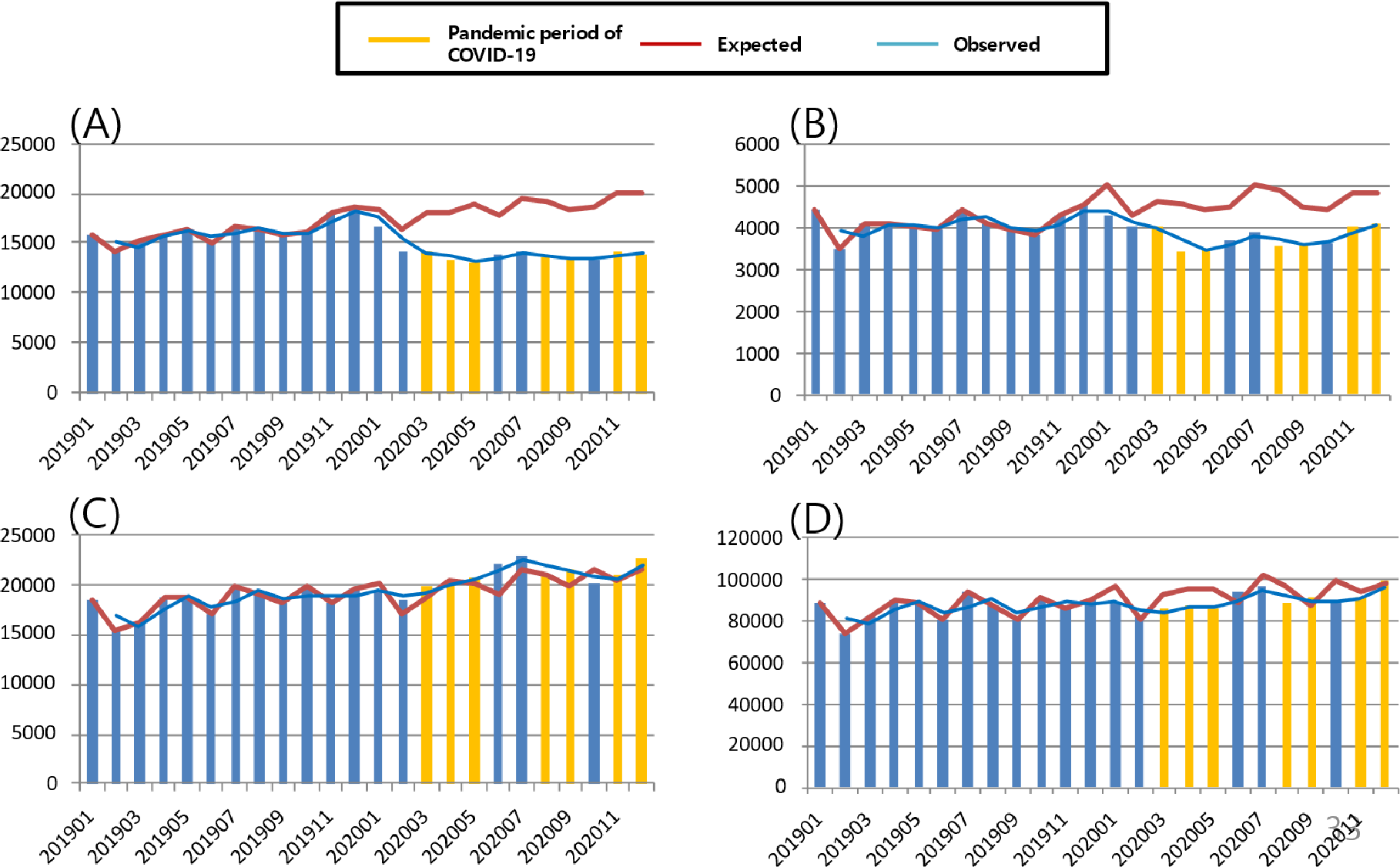

Supplement: S8 Fig — (A) Breast admission, (B) Thyroid admission, (C) Pancreatic outpatient, (D) Lung outpatient. (TIF) [file pone.0296808.s009.tif]

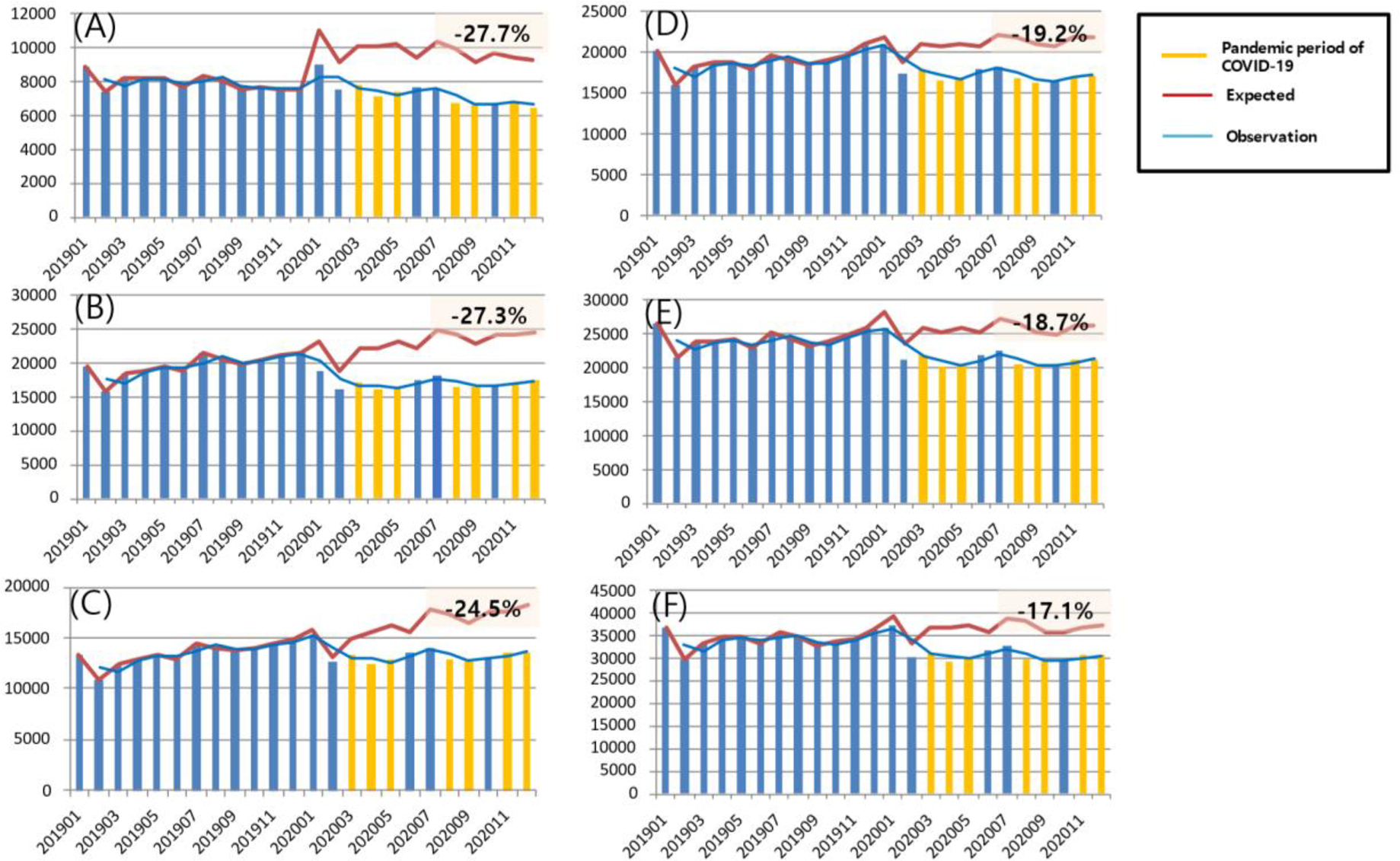

Supplement: S9 Fig — (A) Total medical benefit, (B) 1st Quartile, (C) 2nd Quartile, (D) 3th Quartile, (E) 4th Quartile, (F) 5th Quartile. (TIF) [file pone.0296808.s010.tif]

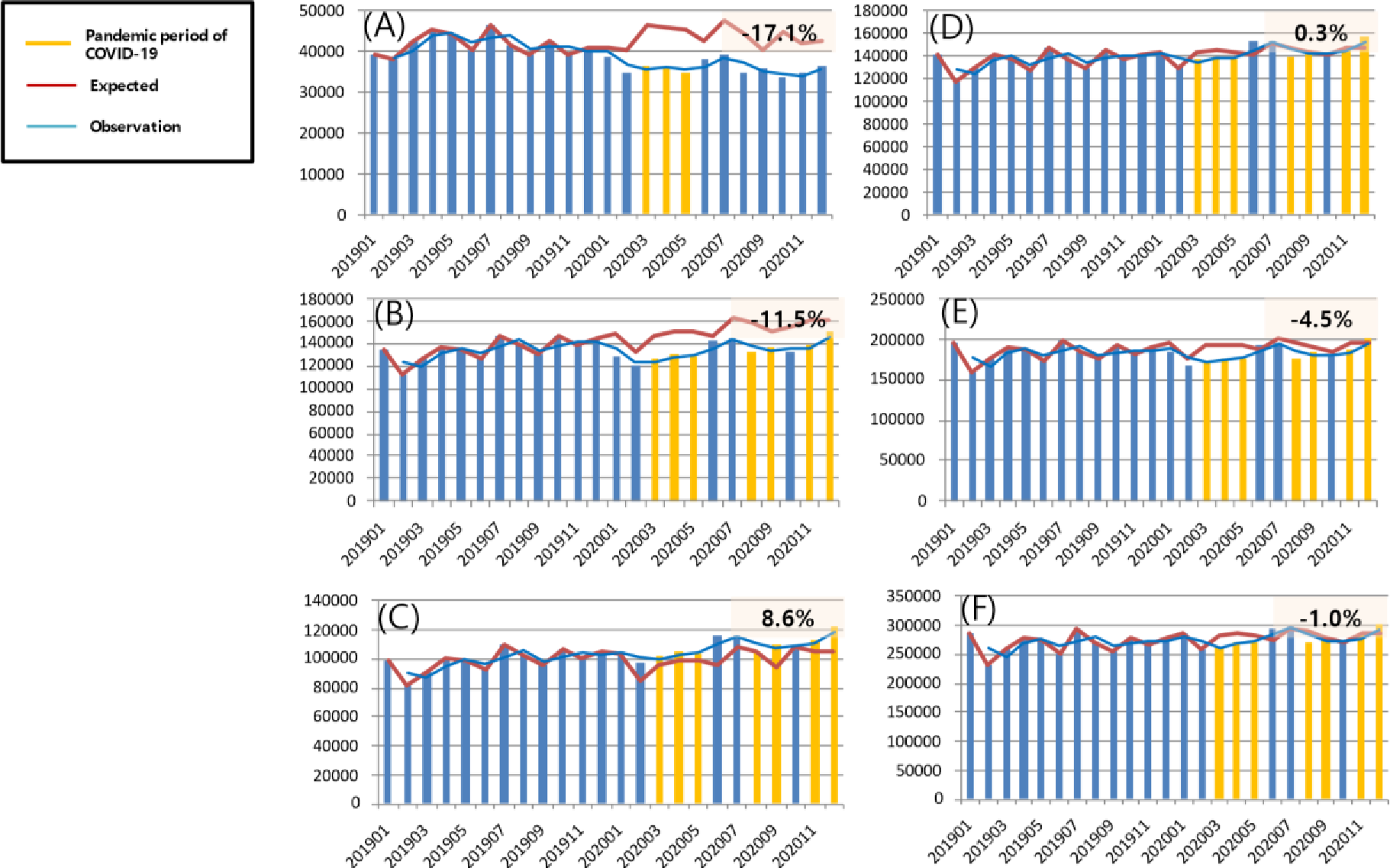

Supplement: S10 Fig — (A) Total medical benefit, (B) 1st Quartile, (C) 2nd Quartile, (D) 3th Quartile, (E) 4th Quartile, (F) 5th Quartile. (TIF) [file pone.0296808.s011.tif]
